# Growth Characteristics of *Chlorella sorokiniana* in a Photobioreactor during the Utilization of Different Forms of Nitrogen at Various Temperatures

**DOI:** 10.3390/plants11081086

**Published:** 2022-04-16

**Authors:** Elvira E. Ziganshina, Svetlana S. Bulynina, Ayrat M. Ziganshin

**Affiliations:** Department of Microbiology, Institute of Fundamental Medicine and Biology, Kazan (Volga Region) Federal University, 420008 Kazan, The Republic of Tatarstan, Russia; elvira.ziganshina@kpfu.ru (E.E.Z.); svsbulynina@stud.kpfu.ru (S.S.B.)

**Keywords:** *Chlorella sorokiniana*, photobioreactor, BBM, anaerobic digester effluent, bacterial community structure, temperature regimens, chemical forms of nitrogen, pigments, proteins

## Abstract

The cultivation of microalgae requires the selection of optimal parameters. In this work, the effect of various forms of nitrogen on the growth and productivity of *Chlorella sorokiniana* AM-02 when cultivated at different temperatures was evaluated. Regardless of the temperature conditions, the highest specific growth rate of 1.26 day^−1^ was observed in modified Bold’s basal medium (BBM) with NH_4_^+^ as a nitrogen source, while the highest specific growth rate in BBM with NO_3_^−^ as a nitrogen source achieved only 1.07 day^−1^. Moreover, *C. sorokiniana* grew well in medium based on anaerobic digester effluent (ADE; after anaerobic digestion of chicken/cow manure) with the highest growth rate being 0.92 day^−1^. The accumulation of proteins in algal cells was comparable in all experiments and reached a maximum of 42% of dry weight. The biomass productivity reached 0.41–0.50 g L^−1^ day^−1^ when cultivated in BBM, whereas biomass productivity of 0.32–0.35 g L^−1^ day^−1^ was obtained in ADE-based medium. The results, based on a bacterial 16S rRNA gene sequencing approach, revealed the growth of various bacterial species in ADE-based medium in the presence of algal cells (their abundance varied depending on the temperature regimen). The results indicate that biomass from *C. sorokiniana* AM-02 may be sustainable for animal feed production considering the high protein yields.

## 1. Introduction

Microalgae are photosynthetic organisms that can be found in various aquatic and terrestrial habitats ranging in size from a few micrometers to several hundred micrometers. Microalgae are already used as food additives and feed in agriculture. These microorganisms require various nutrients for growth and reproduction, including inorganic or organic sources of carbon, nitrogen, phosphorus, sulfur, and iron. The growth efficiency of green microalgae depends both on the composition and pH of nutrient media and on various factors, such as temperature, light sources, and photon flux intensity [1,2].

An important nutrient for microalgae is nitrogen, which is crucial for cell growth and the synthesis of proteins, nucleic acids, and chlorophyll molecules [3]. Microalgae can utilize various forms of nitrogen, such as inorganic nitrate, nitrite, ammonium ions, and organic nitrogen, including urea and amino acids. Nitrate, nitrite, and ammonium ions are the most common sources of inorganic nitrogen used by eukaryotic microalgae. Ammonium, as a reduced form of nitrogen, is one of the cheapest sources of inorganic nitrogen for cultivation of microalgae. Unlike NO_3_^−^, when NH_4_^+^ is utilized, microalgae spend less energy on its assimilation, and NH_4_^+^ is directly incorporated into amino acids [4,5,6]. Although NH_4_^+^ is usually rapidly utilized and preferred over other forms of nitrogen, it is toxic to microalgae and can cause growth inhibition or cell death at elevated concentrations. The results of studies on the resistance of microalgae to ammonium have shown that toxicity may vary depending on the species of microalgae and their nutrient requirements [5,6]. The influence of the specific chemical forms of nitrogen on the metabolic properties of microalgae is often co-dependent on several other factors, such as nutrient supply, temperature, and photon flux intensity [7,8,9].

Temperature is an important factor determining the growth rate of algae, cell size, respiration, biomass production, and the structure of cellular components of microalgal cultures [2]. The temperature regimen affects the enzymatic activity, membrane fluidity, the efficiency of the electron transport chain, and other metabolic mechanisms. Temperature below the optimum limits the growth rate of microalgae, affecting the kinetics of cellular enzymatic processes [10,11]. Exceeding the limiting temperature reduces the efficiency of photosynthesis, since some proteins involved in photosynthesis are deactivated, which leads to a decrease in the productivity of microalgae and even to cell death [12,13]. The optimum growth temperature for the most used microalgae, such as *Chlorella*, *Chlamydomonas*, *Botryococcus*, *Scenedesmus*, *Neochloris*, *Haematococcus* (*Chlorophyta*), and *Nannochloropsis* (*Ochrophyta*), lies within a wide temperature range between 20 °C and 30 °C and depends on the strain [14]. Temperature regimens also affect the quality of pigments and lipids [15,16].

*Chlorella* is one of the important genera of the phylum *Chlorophyta* for the production of biomass and biological compounds (lipids, proteins, carbohydrates, pigments, and vitamins) and application in wastewater treatment [17,18]. *Chlorella sorokiniana*, as one of the promising species of the genus *Chlorella*, can efficiently grow auto-, hetero-, and mixotrophically in a wide temperature range and can be used to develop efficient wastewater treatment technologies [19,20,21].

Fast-growing and highly tolerant microalgae are crucial for various biotechnologies; however, detailed information on the growth and productivity of different strains of microalgae when cultivated in the presence of various forms of nitrogen and at various temperatures is limited. The cultivation of microalgae in wastewaters and effluents from biogas reactors as nutrient media increases the sustainability of microalgae-based biotechnologies. However, municipal, industrial, and agricultural liquid wastes for microalgae cultivation can contain various forms of nitrogen and inhibiting compounds that need to be considered when introducing promising microalgae into biological wastewater treatment systems [5,11,21,22]. Anaerobic digester effluent (ADE) is often characterized by high ammonium/ammonia content, turbidity, and dark color [23,24], which may be the reason for the inhibition of microalgal growth. Despite the attractiveness of using liquid waste for cultivation of microalgae, the effect of waste properties on microalgal growth is still poorly understood. In addition, cultivation of microalgae in wastewater is associated with a high risk of low or uneven productivity and quality of the resulting biomass, often due to contamination with heterotrophic microorganisms. Thus, it is necessary to better understand the effect of nitrogen forms and temperature on the growth and photosynthetic characteristics of promising microalgae, including those in bacterially contaminated wastewater.

In this context, this study aimed to analyze the effect of temperature regimens (30 °C, 33 °C, and 36 °C) on the ability of the microalga *Chlorella sorokiniana* AM-02 to utilize NO_3_^−^ and NH_4_^+^ from a synthetic medium and NH_4_^+^ from an unsterilized effluent from biogas reactors for the accumulation of biomass, pigments, and proteins. The present study also investigated specific bacterial partners of *C. sorokiniana* during cultivation in agricultural wastes at different temperatures.

## 2. Results and Discussion

### 2.1. Growth Parameters of C. sorokiniana AM-02

The main goal of cultivation of microalgae is to reduce the cost of cultivation and time during which the maximum biomass/value products are accumulated. Therefore, it is important to select conditions under which nutrients are readily available and which will provide cost savings for large-scale cultivation. Considering that nitrogen is one of the most important nutrients affecting the biomass characteristics of various microalgae species [5,6], in this study, we compared the effects of various forms of nitrogen at the same initial nitrogen concentration on the growth and productivity of *Chlorella sorokiniana* AM-02 at different temperatures—30 °C, 33 °C, and 36 °C. 

Bold’s basal medium (BBM) [25] and NH_4_^+^-containing liquid phase of digestate after anaerobic conversion of nitrogen-rich chicken and cow manure (anaerobic digester effluent, ADE) were tested on the algal growth. On one hand, when cultivated in a medium with NH_4_^+^, microalgae assimilate ammonia and generate H^+^ ions, which lead to a decrease in pH. On the other hand, when algae grow in a nitrate-containing medium, pH gradually increases until nitrogen is completely depleted [6]. Therefore, in this work, to avoid the effect of pH on growth characteristics, pH was controlled and maintained at a neutral level (7.0 ± 0.05) in all experiments.

**Figure 1:**
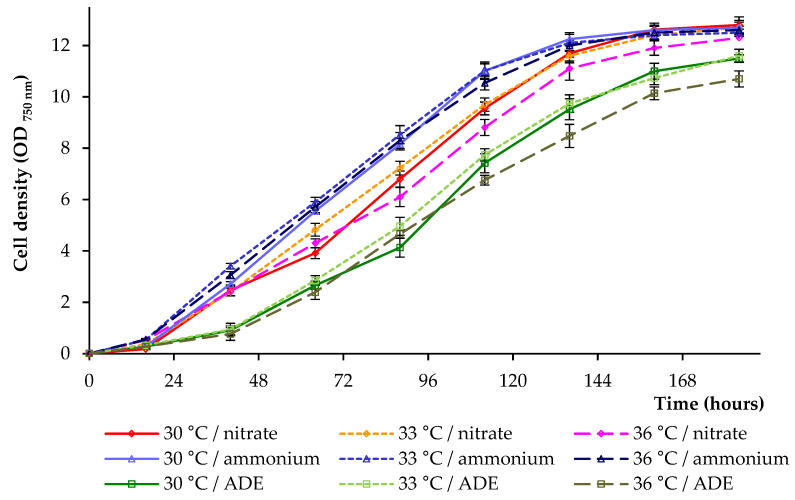
Growth of *C. sorokiniana* AM-02 cultured under different conditions.

**Figure 2:**
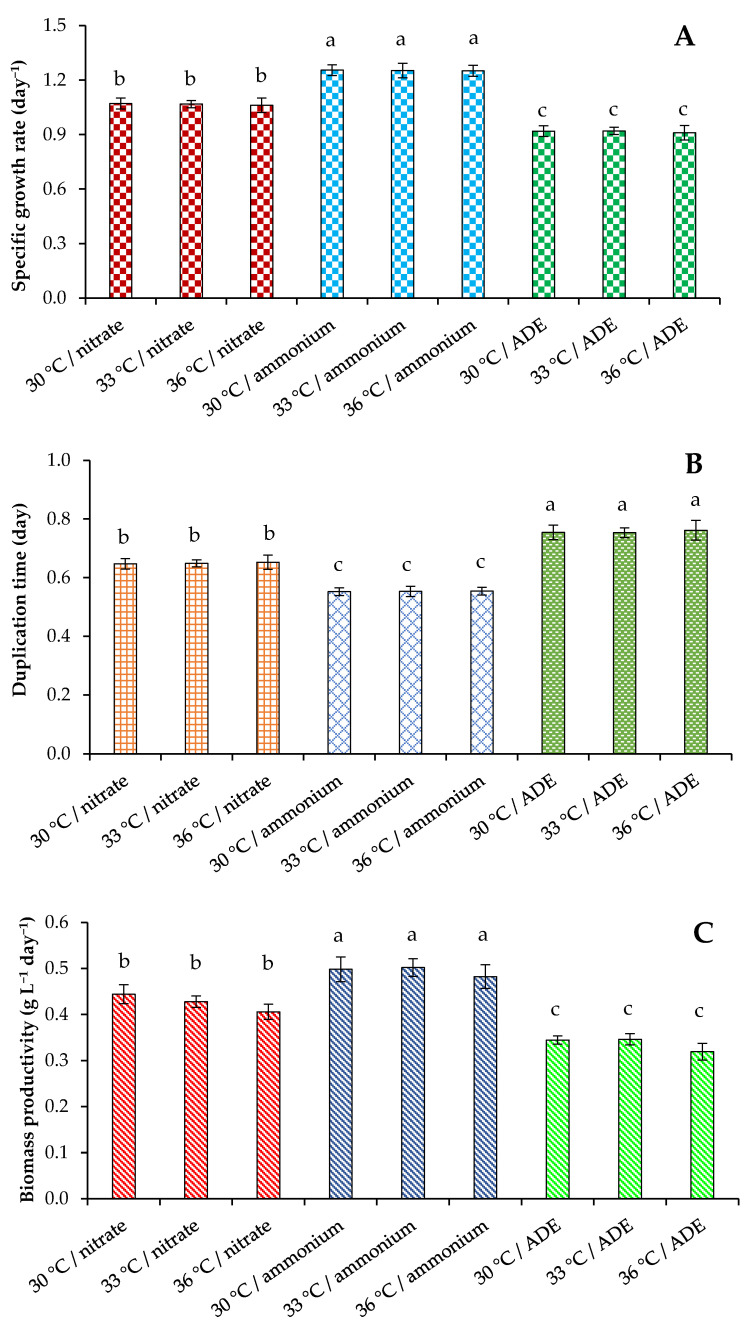
Specific growth rate (**A**), duplication time (**B**), and biomass productivity (**C**) of cultures of *C. sorokiniana* AM-02 grown under different strategies. Means that do not share a letter are significantly different.

Microalga was grown in a sterilized Labfors 4 Lux photobioreactor (Infors HT, Bottmingen, Switzerland). The influence of two chemical forms of inorganic nitrogen and ammonium sources on the growth of *C. sorokiniana* was evaluated based on the analysis of optical density (OD_750_) (Figure 1). Since OD_750_ also measures the growth of bacteria (in ADE-containing treatments), the number of algal cells was additionally counted. Culture characteristics, such as dry weight, volatile solids, and maximum pigments are presented in Table 1, whereas indicators of specific growth rate, duplication time, and biomass productivity are illustrated in Figure 2.

The high values of OD_750_ were noted at 136–184 h of the experiments, and the high specific growth rates were noted when the studied strain was cultivated in a modified medium with NH_4_Cl as the only source of nitrogen (compared to NaNO_3_-containing treatments and ADE-containing treatments). The growth curves, specific growth rate, doubling time, and biomass productivity in these experiments did not show significant differences at 30 °C, 33 °C, and 36 °C (within each block of experiments) (Figure 1 and Figure 2). Final OD_750 nm_ and dry weight biomass values (when grown in the presence of NH_4_Cl or NaNO_3_) were also comparable, whereas volatile solids values obtained in treatments at 36 °C were lower compared to those received at other temperatures (Table 1). The growth of *C. sorokiniana* AM-02 in ADE-based medium demonstrated the lowest OD_750 nm_ with the lowest values obtained at 36 °C (–16.4% on 184 h, when the maximum final OD_750_ values were compared) (Figure 1). Finally, the cultures increased in mean OD_750_ values and cell number to 10.70–12.80 and 3.7–4.2 × 10^8^ cells mL^−1^, accordingly (depending on the treatment).

Significant differences in specific growth rates, duplication times, and biomass productivity between the three sets of experiments were found (Figure 2). Thus, the specific growth rate of the strain *C. sorokiniana* AM-02 reached its maximum value of 1.26 day^−1^ when the alga was grown in modified BBM with NH_4_^+^, 1.07 day^−1^ when the alga was grown in BBM with NO_3_^−^, and 0.92 day^−1^ when the alga was grown in ADE-based medium (Figure 2A). Microalgae cells expend energy to reduce NO_3_^−^ and produce nitrate and nitrite reductases [5], which was probably reflected in a decrease in specific growth rates (when compared to ammonium-containing BBM).

The maximum values of the duplication time achieved 0.55, 0.65, and 0.76 day in experiments with modified BBM containing NH_4_Cl, with BBM containing NaNO_3_, and with ADE medium, respectively (Figure 2B). The biomass productivity values are illustrated in Figure 2C. The productivity values in experiments with *C. sorokiniana* cultivation in synthetic medium with NH_4_^+^ were significantly higher than productivity values observed in treatments with NO_3_^−^ and ADE. Temperature (30–36 °C) did not significantly affect these parameters. Finally, we assume that cultivation of the strain AM-02 in media with ammonium chloride is more economically advantageous.

Considering that NH_4_^+^ is quickly converting to amino acids [5], the increased growth and productivity of the studied green microalga in synthetic medium with NH_4_^+^ can be explained by lower cell energy consumption. Tolerance to ammonium/ammonia differs in different classes of microalgae, and chlorophytes are much more resistant to high levels of ammonium than other unicellular algae [6]. It can be assumed that the strain AM-02 could adapt to high levels of this form of nitrogen in its natural environment. Based on the data of experiments conducted at 30 °C, 33 °C, and 36 °C with NH_4_^+^-containing BBM and ADE, we concluded that 30 °C and 33 °C are the most favorable for the growth of *C. sorokiniana* AM-02.

### 2.2. CO_2_ and Nitrogen Uptake by C. sorokiniana AM-02

**Figure 3:**
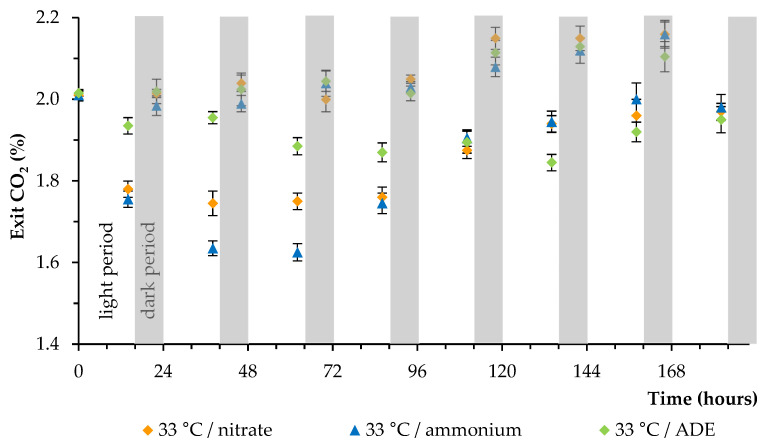
Changes in the level of carbon dioxide released from the photobioreactor under selected conditions (at 33 °C; measured with the Infors gas analyzer).

In all experiments, the culture of microalga was continuously supplied with CO_2_ (800 mL of air containing 2.0% (*v*/*v*) CO_2_ per 1 min). A decrease and an increase in the CO_2_ level from the photobioreactor were observed during the light period and the dark period under all conditions, respectively (Figure 3 demonstrates data obtained at 33 °C). A higher level of initial carbon dioxide uptake by algal culture was observed when grown in BBM in the presence of NH_4_^+^. The lowest level of carbon dioxide uptake by alga was observed when grown in the ADE-based medium.

**Figure 4:**
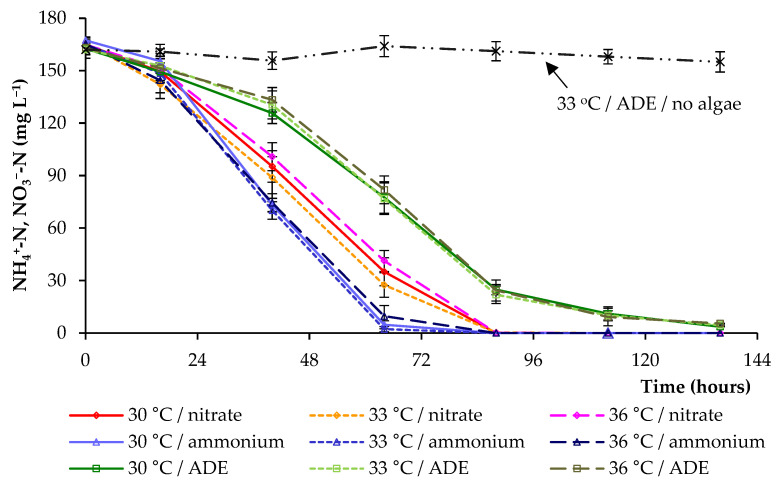
Nitrogen removal by *C. sorokiniana* AM-02.

Anaerobic effluent is often characterized by a high content of ammonia, which accumulates during the anaerobic digestion process [23,26]. The nitrogen removal under different conditions is shown in Figure 4. The concentrations of NH_4_^+^-N and NO_3_^−^-N gradually decreased. The most effective nitrogen consumption was revealed in experiments with NH_4_^+^ in synthetic medium at all temperatures (30 °C, 33 °C, 36 °C). When cultivating microalga in ADE-based medium, a delay of NH_4_^+^-N consumption was observed (Figure 4), which affected the growth parameters and final biomass yield. The results obtained indicated that the temperature had no significant effect on the nitrogen removal (within each block of experiments); however, at 36 °C, the nitrogen consumption was slightly slower in all variants.

The results indicated that NH_4_^+^ from modified BBM was almost completely consumed by *C. sorokiniana* after 64 h, while NH_4_^+^ from ADE was only consumed by *C. sorokiniana* only after 136 h. The removal efficiency of NO_3_^−^-N reached 100% on 88 h. The results showed that distinct components in ADE, along with the dark color of the effluent, reduced the growth of *C. sorokiniana* and limited the removal of NH_4_^+^-N from the culture medium. The results of additional experiments (in the absence of algal cells) aimed at determining the possibility of utilizing a part of NH_4_^+^ by the residual bacteria present in ADE demonstrated the absence of bacterial growth and NH_4_^+^ consumption by bacteria (Figure 4). This led to the conclusion that most of the NH_4_^+^ was utilized by algae. However, during the growth of algal cells and providing nutrients to bacteria (in ADE treatments), part of NH_4_^+^ could additionally be used by bacterial cells.

The tested anaerobically digested agricultural wastes with ammonium as the main form of nitrogen, despite the reduced algal growth performance, represent a cheap source for cultivation of microalgae. The use of these agricultural wastes can reduce costs of cultivation of microalgae and negative environmental impacts.

### 2.3. Pigments and Proteins Accumulation by C. sorokiniana AM-02

**Figure 5:**
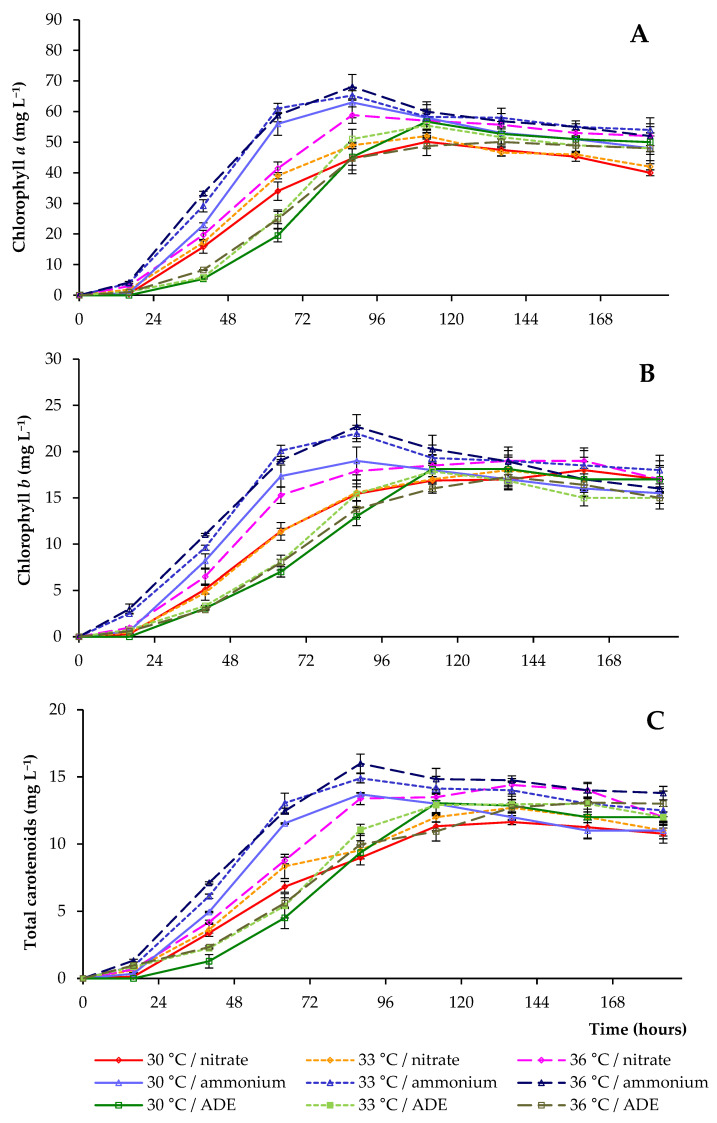
Pigment concentration (chlorophyll *a* (**A**), chlorophyll *b* (**B**), and total carotenoids (**C**)) for cultures of *C. sorokiniana* AM-02 cultured under different conditions.

Figure 5 and Table 1 show that in experiments with NH_4_^+^-N or NO_3_^−^-N in synthetic media the concentrations of chlorophylls *a*, *b,* and total carotenoids at 30 °C were lower than those detected at other temperatures (when the same blocks of experiments were compared). The maximum average concentrations of pigments were 106.8 and 93.9 mg L^−1^ in experiments carried out at 36 °C in synthetic media with NH_4_^+^ and NO_3_^−^ as sole nitrogen sources, respectively (Table 1). Therefore, the presence of NH_4_^+^, as well as an increase in temperature, stimulated the rapid accumulation of pigments in BBM.

The content of chlorophyll *a* in cells increased from the day of inoculation to 88 h, reaching 68.1 and 58.9 mg L^−1^ at 36 °C in NH_4_^+^ and NO_3_^−^-containing media, respectively (Figure 5A). Chlorophyll *b* lines had similar patterns to chlorophyll *a* lines in these treatments, and its level reached 22.7 and 17.9 mg L^−1^ in NH_4_^+^ and NO_3_^−^-containing media, accordingly (Figure 5B). At lower temperatures, chlorophyll levels (when the same blocks of experiments were compared) were lower. After the maximum peaks, a decrease in the concentration of pigments was observed in all media containing different nitrogen sources.

The maximum concentration of pigments in ADE-based media was only noted after 112 h of cultivation. We also showed a substantial decrease in the pigment content in the strain *C. sorokiniana* AM-02 when it was grown in ADE-based medium (when compared to the growth of cells in modified BBM with NH_4_^+^). The maximum values of pigments were in the range of 80.4–87.9 mg L^−1^ (Table 1). This decrease in pigment level was likely mediated by a decrease in light penetration through a dark-colored ADE-based medium and was one of the reasons for the decrease in the rate of photosynthesis. In addition, when algal cells were cultivated in ADE-based medium at 36 °C, a lower yield of pigments was detected. This can be explained by the reduced growth of *C. sorokiniana* under these conditions.

Chlorophyll is an available intracellular pool of nitrogen that is used by algae to support further cell growth and biomass production as the nitrogen reserves in the culture media are depleted [27]. After the depletion of nitrogen in the medium, we noted a decrease in the levels of total chlorophyll in cells, that is, its decomposition allowed microalga to support further cell growth and biomass production (Figure 1, Figure 4 and Figure 5).

**Figure 6:**
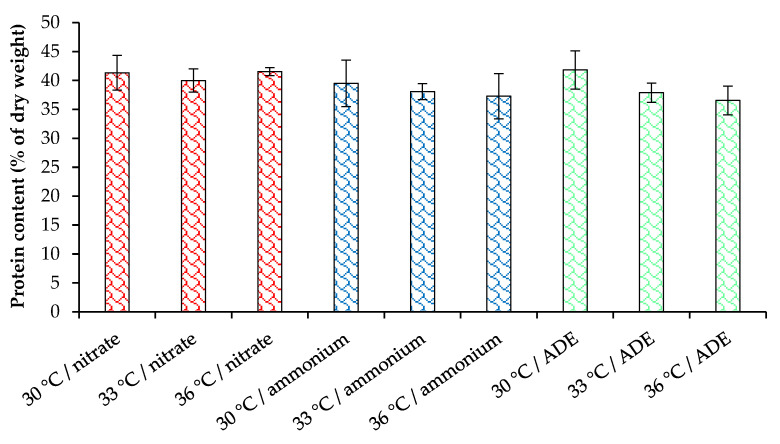
Protein content in cells of *C. sorokiniana* AM-02 cultured under different conditions. No significant differences were observed between treatments.

Considering that nitrogen is one of the main components of protein, we estimated the protein content in the cells of *C. sorokiniana* AM-02 cultured under different conditions (Figure 6). The cultivation of *C. sorokiniana* in synthetic media with NO_3_^−^ and NH_4_^+^ allowed us to obtain a high biomass concentration containing up to 41.4% of protein, while in ADE-containing treatments, the final biomass concentration was lower, but the protein content was at the same level (maximum 41.8% of the dry weight). For microalgae of the genus *Chlorella*, the optimal cultivation temperature is in the range from 28 °C to 35 °C, above which the production of metabolites decreases [28]. Increasing the temperature up to 36 °C did not significantly affect the protein content in our experiments. This research indicates that *C. sorokiniana* AM-02 with high protein content can be suitable for animal feed application.

### 2.4. Comparison of C. sorokiniana AM-02 Growth Characteristics

Algal biotechnology is considered a promising area for extracting nutrients from agricultural residues or wastes. The cultivation of microalgae in anaerobically treated agricultural waste materials solves several problems, such as extracting nutrients from wastes to obtain valuable biomass and reducing energy costs in treatment processes [29]. To increase the growth of microalgae in wastewater, it is necessary to reduce the toxicity of the medium and ensure the availability of nutrients necessary for the growth of algae.

Our recent work has shown that effluent after anaerobic digestion of cattle manure, distiller grains with solubles, and sugar beet pulp can be used as a good nutrient medium for growing *C. sorokiniana*. Additionally, we developed a method for efficient cultivation of microalgae in digestate by adjusting the levels of phosphorus and sulfur and diluting the digestate [21]. We found that the highest growth rate was obtained at 10% effluent loading (1.45 day^−1^), whereas the highest biomass productivity was obtained in experiments with 20% effluent loading (0.50 g L^−1^ day^−1^). The higher specific growth rate and biomass productivity in modified BBM with NH_4_^+^ in our recent study can be explained by the higher values of photosynthetic photon flux density and CO_2_ supply [21].

In the present work, in one of the series of experiments, anaerobically treated unsterilized nitrogen-rich chicken manure and cow manure were used as a nutrient medium for cultivation of algae, additionally supplying them with phosphate and sulphate ions. Stable growth of microalgae in culture medium with ammonium was also achieved by maintaining a neutral pH. In addition, to assess the possible uptake of NH_4_^+^ by bacteria (from an unsterilized effluent), a control experiment was performed in which the effluent medium was not inoculated with *C. sorokiniana* cells. The cultivation in a nutrient medium based on unsterilized wastes can have a beneficial effect on the growth of microalgae, which is also associated with the symbiotic interaction of algae and bacteria, which increases the resistance of algae to inhibitory substances [30,31]. A serious problem is the pollution of the environment by various inhibitory compounds [32].

We found that *C. sorokiniana* AM-02 produced higher biomass values at 30 °C and 33 °C in synthetic media, while a further increase in temperature slightly reduced the biomass yield and productivity (but these data were statistically insignificant). *C. sorokiniana* is a thermotolerant species, and previous findings demonstrated that the growth can be obtained at temperatures over a wide range, 25–40 °C [19]. Several previous studies have also shown that microalgal growth rate, biomass productivity, and the composition of metabolic products depend on the growth conditions and nutrient media characteristics. The effect of temperature regimens on the photosynthetic activity of several microalgae strains, including *Chlorella* species, was previously evaluated [33]. It was shown that the optimum of photosynthesis is in the range from 27.5 °C to 33.3 °C. In another study, Converti et al. [34] investigated the effect of temperature and nitrogen concentrations on the growth of *C. vulgaris* and found that the growth rate decreases above 30 °C. Further temperature increase (38 °C) led to a sharp cessation of the growth and then to cell death. Phototrophic metabolic activity is inhibited at high temperatures, which is reflected in a decrease in growth rate. The key effect of temperature on photosynthesis is due to a decrease in the activity of ribulose-1,5-bisphosphate, the activity of which increases with increasing temperature, but only to a certain level [2,35].

Based on the previous works in this field, it can be concluded that nitrogen can be supplied in any form to stimulate the growth of microalgae, and microalgae have different requirements for the form and concentration of nitrogen [36,37,38,39,40]. Feng et al. [38] evaluated the effect of various nitrogen forms (NaNO_3_, CO(NH_2_)_2_, and NH_4_Cl) and different initial concentrations of N in modified BG-11 (blue-green) medium on the growth of *Chlorella* sp. GN1 in flat plate photobioreactors. The authors showed that the growth of *Chlorella* sp. GN1 was significantly better in media with organic nitrogen and nitrate nitrogen. In addition, it was noted that algal cell growth was inhibited by increasing NH_4_Cl concentration. Nayak et al. [39] also tested different forms of nitrogen (CO(NH_2_)_2_, NaNO_3_, KNO_3_, NH_4_NO_3_, NH_4_Cl, NH_4_CH_3_CO_2_, (NH_4_)_2_SO_4_, or NH_4_HCO_3_) on the growth of *Chlorella* sp. HS2. The authors found a very low biomass productivity during cultivation in BG-11 medium enriched in NH_4_Cl, NH_4_NO_3_, and (NH_4_)_2_SO_4_. However, the results of both studies were obtained without pH control, and the utilization of high NH_4_^+^ concentrations probably resulted in H^+^ release, pH decrease, and growth inhibition. Zheng et al. [40] cultivated *C. vulgaris* in manure-free piggery wastewater with different ammonium content (220, 110, 55, and 27.5 mg L^−1^) at pH 7.0 and determined the optimal NH_4_^+^ concentration for microalgal growth, namely 110 mg L^−1^. Since the preferred source of nitrogen differs for different species of microalgae, research is needed to select the optimal source and concentration of nitrogen.

### 2.5. Bacterial Community Structure in ADE-Containing Growth Medium

While axenic cultures of algae are required for some research and industrial applications, large-scale microalgal biomass production and algae-based bioremediation processes may encounter bacterial invasions. Despite the active introduction of green microalgae into wastewater treatment systems, the role of bacteria in such systems is often ignored, and only a limited number of studies on microbial composition in microalgae production systems have been published. Among them, we note works focused on the simulated co-cultivation of algae and bacteria to assess the impact of both single bacterial species and bacterial communities of water samples on the growth and productivity of microalgae [41,42,43].

Previous studies have shown that the role of bacteria in association with microalgae can be both positive and negative. Thus, researchers suggest that individual bacterial partners of cultures of algae contribute to the increased biomass production and health of microalgae. This increases the efficiency of certain biotechnologies [42,43]. In this study, traditional microbial culture methods have been used to identify the culturable bacteria in ADE-based medium. In addition, the bacterial community structure of the ADE-based growth medium was determined by high-throughput sequencing of the 16S rRNA gene amplicons. The obtained data were analyzed to determine the influence of the temperature regimen on the composition of aerobic bacterial communities during the growth of *C. sorokiniana* in agricultural wastes.

Aerobic bacterial isolates were obtained from ADE-based medium (at the end of cultivation of *C. sorokiniana*) using nutrient agar medium. Isolates were then identified based on morphological, biochemical characteristics, and their taxonomic affiliation was confirmed by the sequencing of their 16S rRNA gene fragments (Table 2).

**Figure 7:**
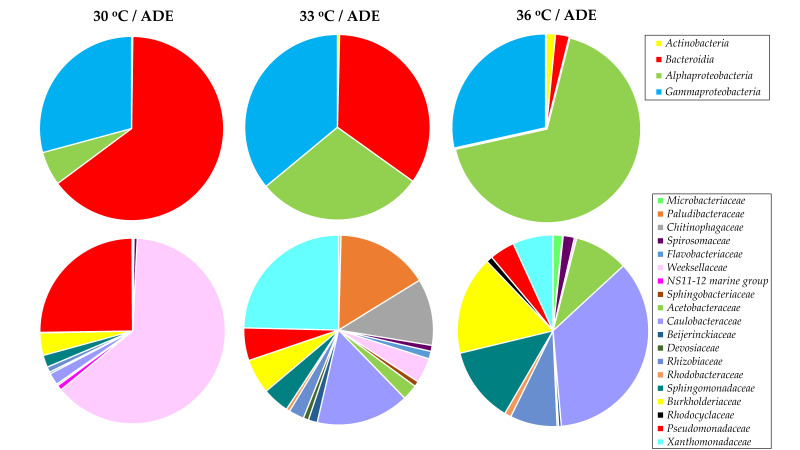
Taxonomic composition of bacterial communities detected during *C. sorokiniana* AM-02 cultivation in ADE-containing media (184 h). Bacterial community structure according to amplicon sequencing of the bacterial 16S rRNA gene is shown on the class (top row) and family (bottom row) levels.

A total of 200 isolates of aerobic bacteria were obtained and 14 16S rRNA gene sequences were determined. All 16S rRNA gene sequences were assigned to the phylum *Proteobacteria* (*Alpha*-, *Beta*- and *Gammaproteobacteria* classes). It should be noted that members of the classes *Alpha*- and *Gammaproteobacteria* made up a large proportion of the communities as determined by high-throughput sequencing of the 16S rRNA gene amplicons (Figure 7). Several 16S rRNA gene sequences of bacterial isolates had low matches in the nucleotide database, and these isolates may represent new bacterial species (Table 2).

Bacterial communities in experiments with ADE were established by analyzing the 16S rRNA gene in samples collected after cultivation of *C. sorokiniana*. A total of 83,943 high-quality bacterial 16S rRNA gene sequences were obtained for 3 samples, and the average number of reads per sample was 27,981 (ranging from 23,129 to 34,476). Sequence analysis showed that *C. sorokiniana* DNA fragments were also amplified with the primers used, and these data were excluded from further analysis.

The diversity of the bacterial communities in ADE-based medium was assessed in terms of alpha diversity. The number of observed operational taxonomic units (OTUs), Chao1 index, and Simpson’s index indicated the difference in bacterial diversity between the samples (Table 3). The observed OTUs, Chao1, and Simpson indexes were slightly higher in the sample from ADE-based treatment with a 30 °C regimen.

Experiments conducted under aeration led to the growth of bacteria belonging to three phyla—*Proteobacteria*, *Bacteroidetes*, and minor *Actinobacteria*. *Firmicutes* (represented by the dominant class *Clostridia*) and *Bacteroidetes* (represented by the dominant class *Bacteroidia*) were noted as the main bacterial phyla in mesophilic anaerobic biogas reactors converting chicken and cow manure, the effluent of which was used in this work [23]. However, most of the detected bacterial species were removed by centrifugation of the digestate.

The composition of the bacterial communities was noted to be temperature sensitive. Thus, with an increase in temperature, the bacteria of the phylum *Bacteroidetes* were depleted and gave way to members of the phylum *Proteobacteria*, which are known to be either facultative anaerobic, aerobic, or even microaerobic [44]. On the class level, species of the *Bacteroidia* were abundant in the sample from 30 °C/ADE treatment (64.6%) and were important in 33 °C/ADE treatment (34.5%), whereas *Alphaproteobacteria*, which can survive on a minimal amount of nutrients, made up 67.6% of the community in 36 °C/ADE treatment (Figure 7).

Most of the bacteria observed in the 30 °C/ADE sample belonged to the families *Weeksellaceae* (63.2%) and *Pseudomonadaceae* (25.1%). Representatives of the families *Xanthomonadaceae* (24.6%), *Caulobacteraceae* (15.9%), *Paludibacteraceae* (15.8%), and *Chitinophagaceae* (11.4%) were detected as important in the sample from 33 °C/ADE treatment. Members of the family *Caulobacteraceae* dominated (35.5%), followed by the families *Burkholderiaceae* (16.6%), *Sphingomonadaceae* (12.8%), and *Rhizobiaceae* (8.0%), in the sample from 36 °C/ADE treatment (Figure 7). The results of another work showed that the composition of the bacterial community affected the efficiency of biomass production and the health of the *C. sorokiniana* strain DOE1412 when cultivated in outdoor reactors [43]. Thus, members of the bacterial orders *Rhizobiales* and *Betaproteobacteriales* demonstrated a positive relationship with algal biomass productivity, whereas members of the order *Chitinophagales* were noted as negative indicators of microalgal culture performance.

**Figure 8:**
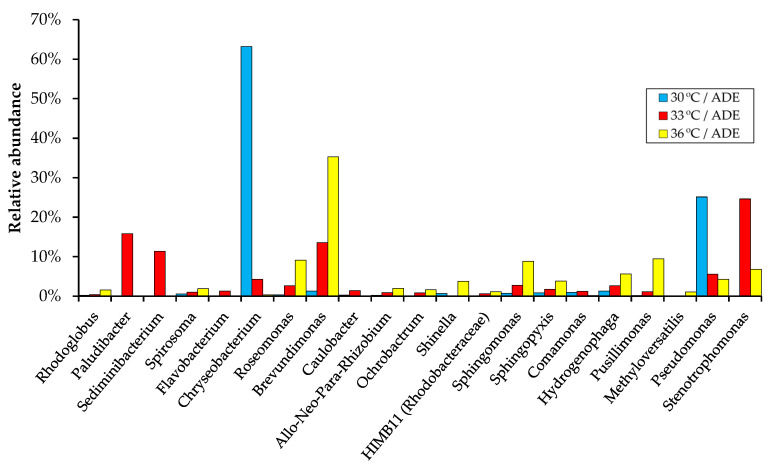
Relative abundance of bacterial genera (based on 16S rRNA gene) detected during *C. sorokiniana* AM-02 cultivation in ADE-containing media (184 h).

Figure 8 shows the composition of bacterial communities on the genus level. So, for each sample obtained after cultivation of algae at a certain temperature, individual representatives of bacterial genera were characteristic. For example, the genus *Chryseobacterium* accounted for 63.2% of the community in the sample from 30 °C/ADE treatment, followed by the genus *Pseudomonas* with the relative abundance of 25.1%. Bacteria of the genera *Brevundimonas*, *Roseomonas*, *Sphingomonas*, and *Pusillimonas* were more abundant in the sample from 36 °C/ADE treatment (their share increased with increasing temperature), whereas the relative abundance of the genera *Chryseobacterium* and *Pseudomonas* dropped sharply (from 63.2% to 0.4% and from 25.1% to 4.3%, respectively).

Bacteria that are beneficial to chlorella include, for example, members of the genera *Brevundimonas* [45] and *Sphingomonas* [46]. It should be noted that the proportion of members belonging to *Allorhizobium–Neorhizobium–Pararhizobium–Rhizobium* clade (*Rhizobiaceae*), described as indicators of the algal health [43], also increased in the samples from ADE-based medium with increasing cultivation temperature (Figure 8). Some species of the genus *Stenotrophomonas* are known to enter into symbiotic interactions with plants [47]. Species of the genera *Chryseobacterium*, *Sphingomonas*, *Brevundimonas*, *Pseudomonas*, and *Hydrogenophaga* are aerobic and widely distributed in the environment, and some species can be involved in bioremediation processes [48,49,50,51,52]. However, some bacterial representatives detected in our systems may cause human infections [53,54,55].

Members of bacterial communities can affect the physiology and metabolism of algae in algae–bacteria associations and determine the effectiveness of biotechnologies. Bacteria contribute to the supply of CO_2_ for photosynthesis and growth factors to microalgae. Nitrogen-fixing bacteria, such as members of the family *Rhizobiaceae*, can provide an additional source of nitrogen for microalgae [56].

It is important to note that in systems for large-scale cultivation of microalgae in non-sterile growth media, monitoring of the bacterial communities is required to achieve high microalgal growth rates and eliminate the risk of stagnation and eventually death of microalgae. The data obtained will complement knowledge about the role of bacteria in artificial systems, from laboratory to industrial, and will help to understand the benefits and risks of complex algal–microbial associations. Thus, the identification of the relationship between algae and bacteria is important not only for obtaining valuable products using cheap nutrient media, such as agricultural wastes, but also has great potential for solving environmental problems.

## 3. Materials and Methods

### 3.1. Microalga

*Chlorella sorokiniana* AM-02 used in this study was isolated from a freshwater lake in Kazan (the Republic of Tatarstan, Russia). The taxonomic status of the culture and some characteristics of its photoautotrophic growth were described by us earlier [57]. The pure culture was maintained on agar plates with standard Bold’s basal medium (BBM) [25] with antibiotics to reduce the risk of bacterial contamination (10 μg and 50 μg ampicillin and kanamycin per 1 mL of medium, respectively). The soil extract and vitamin mix were not added to the original BBM.

### 3.2. Cultivation Conditions

The experiments were divided into three main blocks: (1) cultivation in BBM with NaNO_3_; (2) cultivation in modified BBM in which NaNO_3_ was replaced by NH_4_Cl; and (3) cultivation in a medium containing anaerobically digested chicken and cow manure. In each block of experiments, different temperatures were tested: 30 °C, 33 °C, and 36 °C.

To start all experiments (nine different strategies), the inoculum for cultivation in a photobioreactor was obtained from colonies transferred under aseptic conditions. The culture was then incubated in 250 mL Erlenmeyer glass flasks containing 30 mL of the original BBM on a shaker at 120 rpm at 30 °C and under 400 μmol photons m^−2^ s^−1^ of artificial light provided by Gro-Lux tubes (when measured on the surface of the flasks) for 5 days. Photon flux intensity was measured using a photosynthetically active radiation meter (PAR meter, Apogee Instruments, Logan, UT, USA). Then, the obtained biomass of the microalgae was separated from the medium by centrifugation at 5000× *g*, washed with sterile K-Na-phosphate buffer (pH 7.0), and added into a photobioreactor with an OD_750_ (optical density at 750 nm) of 0.01 at time zero.

Microalgae were grown in a sterilized 3.6 L Labfors 4 Lux photobioreactor (Infors HT, Bottmingen, Switzerland) with a working volume of 2.6 L. The photobioreactor was illuminated with Gro-Lux tubes with high blue and red radiation. The experiments in the photobioreactor were carried out under three temperature conditions, namely 30 °C, 33 °C, and 36 °C. The reactor was continuously stirred at a speed of 120 rpm. For all experiments, a constant high illumination (1000 μmol photons m^−2^ s^−1^; measured on the surface) with a 16:8 light/dark cycle and bubbling of atmospheric air containing 2.0% (*v*/*v*) carbon dioxide were maintained. Aeration (800 mL per 1 min) was provided by a compressor through a 0.45 μm filter, whereas the addition of carbon dioxide was provided by a thermal mass flow controller (Vögtlin Instruments, Aesch, Switzerland). All experiments were conducted with controlled pH throughout the whole experimental period (pH 7.0 ± 0.05). pH was measured with an EasyFerm Plus PHI K8 200 electrode (Hamilton, OH, USA). 8% NaOH or 4% HCl were used to maintain the desired pH of the medium. When observing a foam, a sterile 2% Antifoam B solution (Sigma-Aldrich, St. Louis, MO, USA) was added. Light, temperature, pH of the medium, the pressure inside the reactor, carbon dioxide flow, and the percentage of oxygen and carbon dioxide released were measured by the different Infors devices and displayed online on a computer screen. To test reproducibility, two independent experiments for each growth strategy were performed, and the results were taken as the average.

### 3.3. Identification of Optimal Nitrogen Form at Various Temperatures

To study the effect of two different forms of inorganic nitrogen and ammonium sources on the growth and productivity of microalgae under different temperature conditions, microalgae were cultivated: (1) in BBM with NaNO_3_; (2) in modified BBM with NH_4_Cl instead of NaNO_3_; (3) and in ADE-based medium containing NH_4_^+^ as the main form of nitrogen. In each block of experiments, temperatures of 30 °C, 33 °C, and 36° C were tested. Nitrogen was not expected to become a limiting nutrient, so NO_3_^−^ in BBM and NH_4_^+^ in modified BBM were provided in excess (~730 mg L^−1^ and ~215 mg L^−1^, correspondingly). The initial concentration of NO_3_^−^-N and NH_4_^+^-N in the growth media in all treatments was 165 ± 2.0 mg L^−1^. All other components in the BBM were at the standard concentration as previously reported [25].

Chicken manure and cow manure for the experiments were collected from local farms (Kazan, the Republic of Tatarstan, Russia). The effluent after mesophilic anaerobic digestion of chicken/cow manure contained a high content of ammonium (2.4–2.6 g L^−1^) and had a rich dark brown, black color [23]. In this regard, before being introduced into the photobioreactor, it was centrifuged twice at 10,000× *g* for 10 min to remove the sediment and diluted with sterile distilled water to reach NH_4_^+^-N at a concentration of 165 ± 2.0 mg L^−1^, as for the second group of experiments. Due to the low concentration of phosphate and sulfate ions in ADE-based medium, K_2_HPO_4_ and H_2_SO_4_ were added to the diluted effluent to achieve standard P/S concentrations as in BBM (~160 mg L^−1^ and ~40 mg L^−1^ for phosphate and sulfate ions, respectively).

### 3.4. Analytical Methods

Samples for analysis of algal growth, pigment concentration, protein content, nitrate, and ammonium concentrations in the culture medium were collected every day.

Algal growth was monitored by measuring optical density at 750 nm using a Lambda 35 spectrophotometer (Perkin Elmer, Singapore). If necessary, samples were diluted to obtain an optical density in the range of 0.1–0.4. The number of cells was additionally determined using a hemocytometer.

The specific growth rate and biomass productivity were calculated according to the equations given by Lizzul et al. [19]. The final biomass yield and the level of volatile solids were analyzed as described previously [21]. The doubling time (the time required to achieve a doubling of the number of viable cells) was calculated according to the equation given by Nayak et al. [58].

The concentrations of photosynthetic pigments, namely chlorophylls *a* and *b*, total carotenoids, were measured by using dimethyl sulfoxide extraction method. In total, 1 mL of culture was centrifuged at 10,000× *g* for 5 min, after which the supernatant was discarded. In total, 1 mL of dimethyl sulfoxide (preheated to 60 °C) was added to the precipitate and then resuspended by vortexing. The suspension was heated at 60 °C for 5 min in a thermoshaker TS-100C (BioSan, Riga, Latvia), followed by centrifugation at 10,000× *g* for 5 min. Absorption was read using the Lambda 35 spectrophotometer (Perkin Elmer, Singapore), and calculations for chlorophylls and total carotenoids were conducted according to the equations reported by Wellburn [59].

For protein content determination, approximately 20 mg of dried biomass was combined with 1 mL of 0.5 M NaOH in a 2 mL metal lysing tube (MP Biomedicals, Illkrich, France) with two types of beads (0.6 g of 0.1 mm zirconium/silica beads and 0.4 g of 1.0 mm glass beads). A FastPrep-24 homogenizer (MP Biomedicals, Solon, OH, USA) was used to homogenize the tubes for two 30 s pulses at speed level 6.0 m s^−1^ followed by a 5 min cooling period between pulses. The extracted samples were incubated at 80 °C for 10 min at 500 rpm on the thermoshaker TS-100C (BioSan, Riga, Latvia) and then allowed to cool to room temperature. The mixtures were then transferred to new clean tubes, centrifuged at 2000× *g* for 5 min, and supernatants were used for protein measurement. Concentration of solubilized protein was quantified by the Bradford dye-binding method (Bio-Rad, Munich, Germany). Values are expressed as weight percent (% weight proteins/weight dry biomass).

Nitrate, phosphate, and sulfate concentrations were measured using a Dionex ICS-900 ion chromatography system (Thermo Fisher Scientific, Wilmington, DE, USA) as previously reported [57]. The concentration of ammonium was determined by using Nessler’s reagent (Sigma-Aldrich, St. Louis, MO, USA) as previously described [21].

The determination was carried out in triplicate, and their average results are given with the standard deviations.

### 3.5. Bacterial Community Structure Analysis

Bacterial isolates were obtained from ADE-based medium (after the growth of *C. sorokiniana* on 184 h) on nutrient agar plates and then cultured on nutrient agar plates at 30–36 °C for 24–72 h. Bacterial 16S rRNA gene fragments were amplified by PCR using the universal primers UniBac27f and Univ1492r. About 200 bacterial colonies were picked up and screened in PCR reactions. The library was then analyzed for restriction fragment length polymorphisms applying the restriction endonuclease *Hae*III (Thermo Fisher Scientific, Vilnius, Lithuania). Restriction patterns were clustered using the Phoretix 1D software v. 16.2 (Nonlinear Dynamics, Newcastle upon Tyne, UK). Representative bacterial isolates from each major cluster were partially sequenced. The sequences were compared to the NCBI database using the BLAST program and taxonomically assigned according to the RDP Classifier.

The composition of the bacterial communities in ADE-based medium (at the end of cultivation of microalgae on 184 h) was also investigated using an Illumina MiSeq sequencing platform. Total DNA was extracted and purified from three samples (from one replicate with each temperature regimen) after centrifugation of 5 mL at 14,000× *g* for 10 min using a FastDNA spin kit (MP Biomedical, Solon, OH, USA), according to the manufacturer’s protocol. Amplification, sequencing of the bacterial 16S rRNA genes on the Illumina MiSeq platform, as well as data analysis, were conducted as described in our previous works [21,60]. Finally, bacterial 16S rRNA gene sequences were clustered into OTUs, and their taxonomic assignment was achieved using the Silva database. In addition, OTUs representing less than 0.1% of the total reads and sequences not belonging to bacteria were excluded from further analysis.

### 3.6. Statistical Analyses

Tukey’s multiple comparison tests (α = 0.05) were used to compare differences (Minitab software version 20.2.0).

## 4. Conclusions

*Chlorella sorokiniana* AM-02 efficiently provides a high level of biomass and can be considered a suitable biological resource. The results also indicate that the growth of microalga in synthetic media slightly depend on the temperature (within a range 30–36 °C). The highest specific growth rate of 1.26 day^−1^ was observed in modified BBM with NH_4_^+^ as a nitrogen source. The highest specific growth rate in BBM with NO_3_^−^ as a nitrogen source achieved 1.07 day^−1^. Biomass productivity values reached 0.41–0.50 g L^−1^ day^−1^ in BBM treatments (depending on condition). In addition, *C. sorokiniana* grew well in ADE-based medium (in digestate after anaerobic conversion of chicken/cow manure), with the highest specific growth rate being 0.92 day^−1^. The highest biomass productivity of 0.35 g L^−1^ day^−1^ was obtained during cultivation in ADE-based medium at 30–33 °C. With an increase in the temperature of cultivation in ADE-treatments, the composition of bacterial communities changed dramatically. The tested alga effectively grows in ammonium-rich wastewater, and its potential can be used to prevent environmental pollution. Finally, microalgal biomass with the high protein content can be suitable as animal feed additives.

## Figures and Tables

**Table 1:** *C. sorokiniana* culture characteristics when grown under different conditions.

Treatment	Final OD (750 nm)	Dry Weight (g L^−1^)	Volatile Solids (g L^−1^)	Maximum Chlorophylls and Carotenoids (mg L^−1^)
30 °C/NO_3_^−^	12.80 ± 0.31 ^a^	2.93 ± 0.14 ^a^	2.80 ± 0.12 ^a^	78.8 ± 2.8 ^c^
33 °C/NO_3_^−^	12.70 ± 0.28 ^a^	2.86 ± 0.08 ^a,b^	2.74 ± 0.08 ^a,b^	82.7 ± 3.5 ^c^
36 °C/NO_3_^−^	12.30 ± 0.16 ^a,b,c^	2.72 ± 0.11 ^a,b^	2.43 ± 0.13 ^b,c^	93.9 ± 4.3 ^a,b,c^
30 °C/NH_4_^+^	12.70 ± 0.21 ^a^	2.83 ± 0.15 ^a,b^	2.64 ± 0.08 ^a,b^	96.3 ± 3.1 ^a,b,c^
33 °C/NH_4_^+^	12.50 ± 0.14 ^a,b^	2.85 ± 0.11 ^a,b^	2.69 ± 0.03 ^a,b^	102.1 ± 5.7 ^a,b^
36 °C/NH_4_^+^	12.60 ± 0.16 ^a^	2.74 ± 0.15 ^a,b^	2.44 ± 0.08 ^b,c^	106.8 ± 4.5 ^a^
30 °C/ADE	11.50 ± 0.14 ^c,d^	2.65 ± 0.07 ^a,b^	2.52 ± 0.07 ^a,b,c^	87.9 ± 3.9 ^b,c^
33 °C/ADE	11.60 ± 0.25 ^b,c,d^	2.66 ± 0.09 ^a,b^	2.53 ± 0.04 ^a,b,c^	86.3 ± 4.8 ^b,c^
36 °C/ADE	10.70 ± 0.31 ^d^	2.46 ± 0.14 ^b^	2.23 ± 0.10 ^c^	80.4 ± 5.5 ^c^

Different superscripts indicate differences between the treatments (ANOVA, Tukey method, α = 0.05). Means that do not share a letter are significantly different.

**Table 2:** Sequencing results of 16S rRNA genes of representative bacterial isolates.

Isolate (bp)	Highest BLAST Hit (Acc. No.)/ Percent Identity	Taxonomic Affiliation according to RDP
BS_A2 (821)	*Herminiimonas glaciei* strain UMB49 (NR_044508)/100.00%	*Herminiimonas* sp.
BS_A4 (875)	*Pseudochrobactrum asaccharolyticum* strain CCUG 46016 (NR_042474)/100.00%	*Pseudochrobactrum* sp.
BS_B1 (886)	*Brevundimonas aurantiaca* strain CB-R (NR_028889)/100.00%	*Brevundimonas* sp.
BS_B3 (836)	*Brevundimonas faecalis* strain CS20.3 (NR_117187)/98.44%	*Brevundimonas* sp.
BS_B6 (814)	*Stenotrophomonas pavanii* strain LMG 25348 (NR_118008)/98.40%	*Stenotrophomonas* sp.
BS_C1 (870)	*Paracoccus solventivorans* strain DSM 6637 (NR_119238)/100.00%	*Paracoccus* sp.
BS_C2 (883)	*Brevundimonas aurantiaca* strain CB-R (NR_028889)/100.00%	*Brevundimonas* sp.
BS_D1 (890)	*Phyllobacterium myrsinacearum* strain NBRC 100019 (NR_113874)/100.00%	*Phyllobacterium* sp.
BS_E6 (826)	*Paracoccus versutus* strain NBRC 14567 (NR_113662)/99.88%	*Paracoccus* sp.
BS_F1 (833)	*Brevundimonas aurantiaca* strain CB-R (NR_028889)/100.00%	*Brevundimonas* sp.
BS_G3 (854)	*Comamonas jiangduensis* strain YW1 (NR_109655)/99.88%	*Comamonas* sp.
BS_H1 (859)	*Stenotrophomonas koreensis* strain TR6-01 (NR_041019)/99.77%	*Stenotrophomonas* sp.
BS_H2 (668)	*Pseudomonas paralactis* strain DSM 29164 (NR_156987)/96.71%	*Pseudomonas* sp.
BS_H5 (776)	*Pseudomonas litoralis* strain 2SM5 (NR_108487)/99.23%	*Pseudomonas* sp.

**Table 3:** Alpha diversity of bacterial communities in ADE-containing experiments.

Treatment	Observed OTUs	Chao1 Index	Simpson’s Index
30 °C/ADE	51	54	0.74
33 °C/ADE	47	47	0.70
36 °C/ADE	46	46	0.66

## Data Availability

The data presented in this study are available on request from the corresponding author. The data are not publicly available as the authors prefer to share the data directly with those interested.
